# Antimicrobial potential and osteoblastic cell growth on electrochemically modified titanium surfaces with nanotubes and selenium or silver incorporation

**DOI:** 10.1038/s41598-022-11804-6

**Published:** 2022-05-18

**Authors:** Kevin Staats, Magdalena Pilz, Jie Sun, Tzvetanka Boiadjieva-Scherzer, Hermann Kronberger, Selma Tobudic, Reinhard Windhager, Johannes Holinka

**Affiliations:** 1 Department of Orthopedics and Trauma Surgery, Medical University of Vienna, Waehringer Guertel 18-20, 1090 Vienna, Austria; 2 Competence Center of Electrochemical Surface Technology (CEST GmbH), Wiener Neustadt, Austria; 3 Institute for Chemical Technology and Analytics, Technical University of Vienna, Vienna, Austria; 4 Department of Internal Medicine I, Division of Infectious Diseases and Tropical Medicine, Medical University of Vienna, Vienna, Austria

**Keywords:** Nanomedicine, Biomedical engineering, Biomedical materials, Bacterial infection, Preclinical research

## Abstract

Titanium nanotube surfaces containing silver, zinc, and copper have shown antimicrobial effects without decreasing osteoblastic cell growth. In this in-vitro study we present first results on the biological evaluation of surface modifications by incorporating selenium and silver compounds into titanium-dioxide (TiO_2_) nanotubes by electrochemical deposition. TiO_2_-nanotubes (TNT) and Phosphate-doped TNT (pTNT) were grown on the surface of Ti6Al4V discs by anodization. Hydroxyapatite (HA), selenium (Se) and silver (Ag) compounds were incorporated by electrochemical deposition. Colony forming units of *Staphylococcus epidermidis (DSM 3269)* were significantly decreased in SepTNT (0.97 ± 0.18 × 10^6^ CFU/mL), SepTNT-HA (1.2 ± 0.39 × 10^6^ CFU/mL), AgpTNT (1.36 ± 0.42 × 10^6^ CFU/mL) and Ag_2_SepTNT (0.999 ± 0.12 × 10^6^ CFU/mL) compared to the non-modified control (2.2 ± 0.21 × 10^6^ CFU/mL). Bacterial adhesion was calculated by measuring the covered area after fluorescence staining. Adhesion was lower in SepTNT (37.93 ± 12%; *P* = 0.004), pTNT (47.3 ± 6.3%, *P* = 0.04), AgpTNT (24.9 ± 1.8%; *P* < 0.001) and Ag_2_SepTNT (14.9 ± 4.9%; *P* < 0.001) compared to the non-modified control (73.7 ± 11%). Biofilm formation and the growth of osteoblastic cells (MG-63) was observed by using Crystal Violet staining. Biofilm formation was reduced in SepTNT (22 ± 3%, *P* = 0.02) and Ag_2_SepTNT discs (23 ± 11%, *P* = 0.02) compared to the non-modified control (54 ± 8%). In comparison with the non-modified control the modified SepTNT-HA and pTNT surfaces showed a significant higher covered area with osteoblastic MG-63-cells. Scanning electron microscope (SEM) images confirmed findings regarding bacterial and osteoblastic cell growth. These findings show a potential synergistic effect by combining selenium and silver with titanium nanotubes.

## Introduction

Periprosthetic joint infection (PJI) still remains as one of the most challenging complications after Total Joint Arthroplasty (TJA) with a dramatical impact on the patients’ morbidity and mortality as well as a socio-economic burden for the public health system^1–6^.

Due to an ongoing increase in antibiotic resistance, many efforts have been made for novel antimicrobial therapeutic approaches^7^. The formation of titanium-dioxide (TiO_2_) nanotubes (TNT) has already gained attention due to their antibacterial and osseointegrative potential on orthopedic-relevant surfaces^8–10^. It has been proposed that TNT reduce bacteria on titanium surfaces by inhibiting bacterial adhesion. This can be achieved due to a higher hydrophilicity and an impaired agglomeration of the bacterial cells due to the tubular structure of the TNT^11^. Another option is to apply a coating with bactericidal properties at the implant surface. The use of bacterial killing ions like silver^12–16^, zinc^17^ or selenium^18–22^ has been investigated and silver coated implants are already commercially available. Furthermore, differently to the antibacterial mechanism of TNT, these metals kill bacterial cells by ion release^10^. This effect can be increased by using nanoparticles of these metals^10^. Additionally, the use of TNT has shown to enhance osteogenic potential^23^ which could also be reported for selenium^21,24^. However, there is some evidence that silver compounds might be able to negatively influence osteogenesis due to cytotoxic effects^25^. Thus, even if some retrospective studies could show a decrease in PJI in high-risk patients, there is not enough evidence at the moment to support the global use of silver-ions on regular arthroplasty patients^26^. A study by Holinka et al.^27^ revealed a decreased bacterial cell growth and biofilm formation of *Staphylococcus aureus and Staphylococcus epidermidis (S. epidermidis*) when selenium is used. However, the use of doping/soaking mechanisms to incorporate selenium on the nanostructured surface makes the selenium deposition less controllable. Hydroxyapatite (HA) is one of the most commonly used additive on cementless implants. The inorganic mineral which consists of a calcium-phosphate-hydroxide compound represents the gold-standard for osseoinductive agents.

With the use of anodization our study group was able to develop a strategy to form uniformly shaped TNT. Furthermore, electrochemical deposition enabled the incorporation of particles (pure metals or compounds thereof) and other beneficial agents (e.g. HA) into/onto the nanotubes to eventually enhance antimicrobial and osseointegrative potential^28,29^. Additionally, we were able to create TNT with a desired width of 100 nm to provide antibacterial properties and osseointegration and provide sufficient space for the deposition of antimicrobial agents in the nanotubes. This method enables the combination of different antimicrobial compositions to enhance synergistic antimicrobial and osseointegrative effects. The present study aimed to investigate the antimicrobial effects of the newly formed surfaces and evaluate the osteoblastic cell growth in an in-vitro study. We compared the bacterial adhesion, biofilm formation and osteoblastic cell growth of selenium and silver doped TiO_2_-nanotubes with regular titanium surfaces and HA-doped TiO_2_-nanotubes.

## Results

### Surface characterization

**Figure 1:**
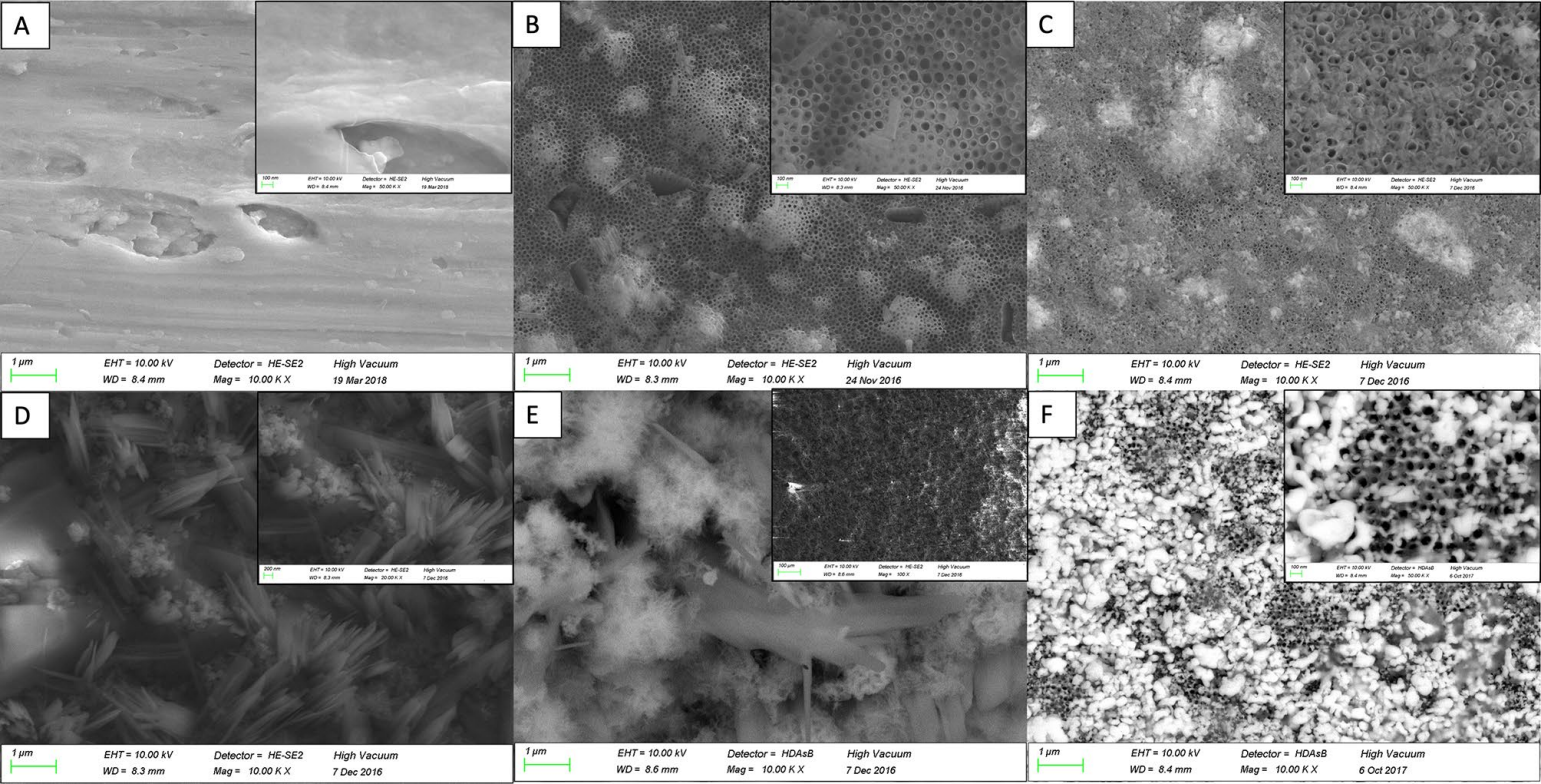
Scanning electron microscope (SEM) images of the discs’ surfaces: (**A**) unmodified control; (**B**) phospate-doped titantium nanotubes (pTNT); (**C**) selenium-incorporated phosphate-doped TiO_2_-nanotubes (SepTNT); (**D**) hydroxyapatite coated (HA) phosphate-doped TiO_2_-nanotubes; (**E**) selenium incorporated phosphate-doped TiO_2_-nanotubes with hydroxyapatite coating (SepTNT-HA); (**F**) silver-selenium incorporated phosphate-doped TiO_2_-nanotubes (Ag_2_SepTNT).

Surfaces of titanium discs with modifications in terms of TNT and pTNT and additional electrochemical deposition of Se, Ag and HA were further investigated regarding surface characteristics. Elemental composition analysis by EDX showed an amount of Se on SepTNT and SepTNT-HA disc surfaces of a mean wt% of 29.9 ± 6.4 wt%. The mean amount of Se and Ag on Ag_2_SepTNT disc surfaces was 17.8 ± 5.4 wt% and 18.8 ± 9.4 wt% respectively. The mean Ag wt% on Ag-pTNT disc surfaces was 6 ± 2 wt%. Visualization of the discs’ surface was carried out via scanning electron microscopy (SEM; ZEISS SIGMA HD VP) equipped with a Schottky field emission source for increased spatial resolution (Fig. 1). Further information regarding surface characteristics and properties from the present surface modification has already been published^28^.

### Bacterial cell adhesion and biofilm formation

**Figure 2:**
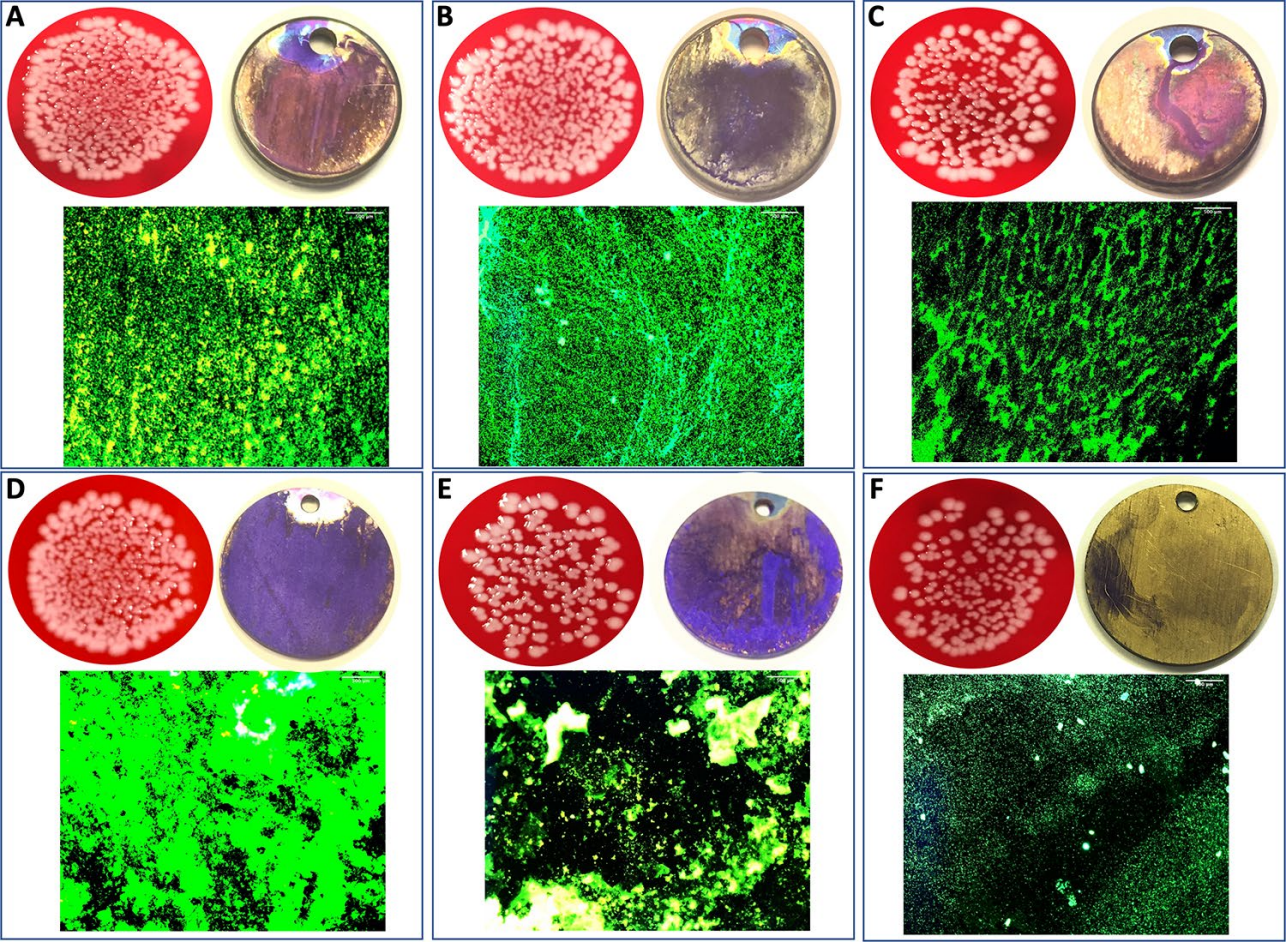
Bacterial cell adhesion and biofilm formation: Bacterial cell count (upper left) was evaluated by culturing *S. epidermidis* on the modified discs. After incubation, discs were sonicated and the supernatant was transferred on agar plates and cultured overnight. CFU/mL were evaluated by counting cells with image processing software. Biofilm formation on titanium discs after incubation with *S. epidermidis* and crystal violet staining (upper right). Biofilm was measured by analyzing the covered area with image processing software. Bacterial cell adhesion was measured after fluorescence staining with SYTO 9 (bottom image) by calculating the covered area. Selenium-silver modified discs exhibited distinct reduction in bacterial cell adhesion and biofilm formation. (**A**) unmodified control; (**B**) phosphate-doped titantium nanotubes (pTNT); (**C**) selenium-incorporated phosphate-doped TiO_2_-nanotubes (SepTNT); (**D**) hydroxyapatite coated (HA) phosphate-doped TiO_2_-nanotubes; (**E**) selenium incorporated phosphate-doped TiO_2_-nanotubes with hydroxyapatite coating (SepTNT-HA); (**F**) silver/selenium incorporated phosphate-doped TiO_2_-nanotubes (Ag_2_SepTNT).

**Figure 3:**
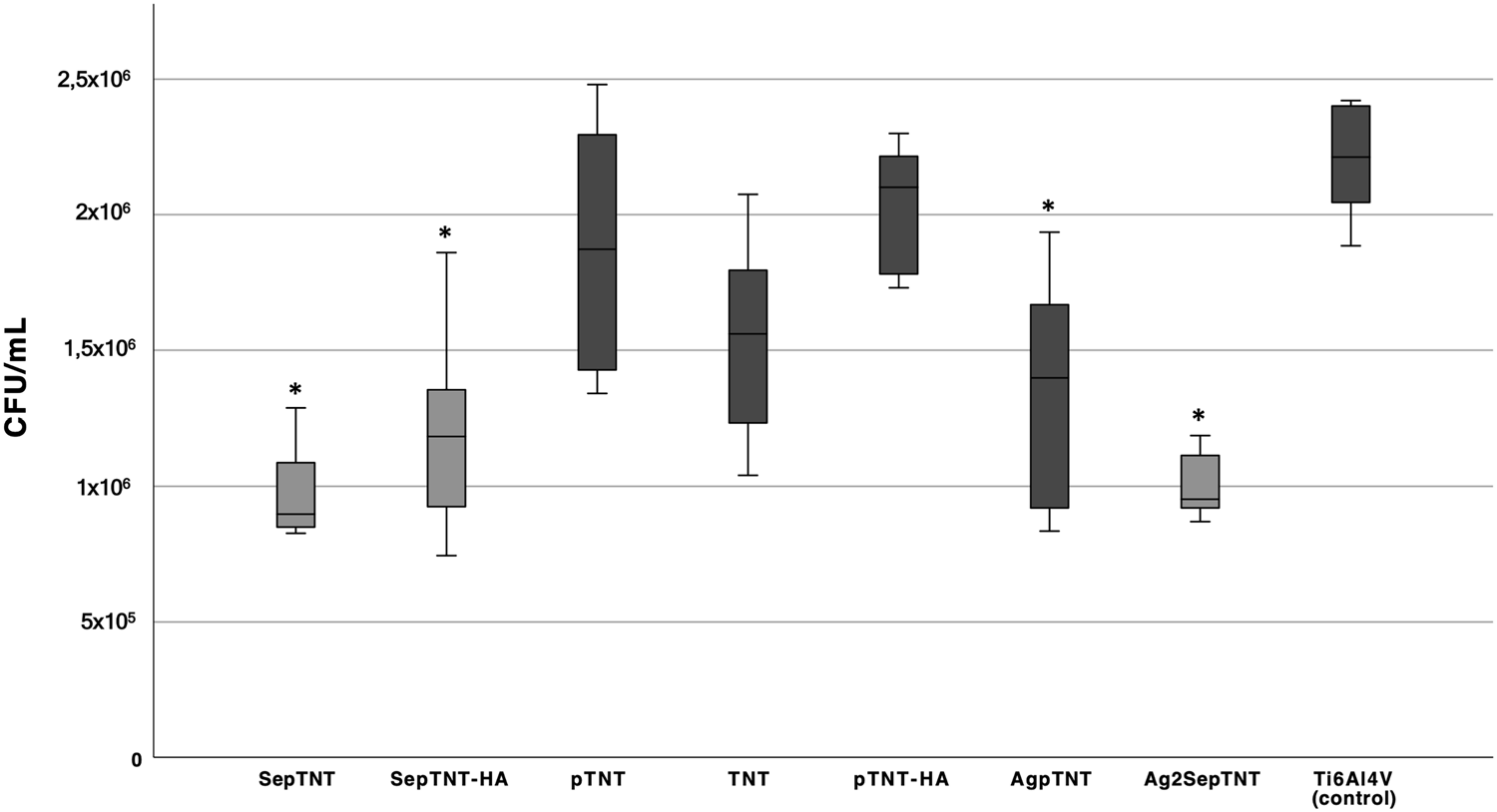
Colony forming units of *S. epidermidis* after 24 h of incubation on the surface of the modified and non-modified (control) titanium discs (*P < 0.05). selenium-incorporated phosphate-doped TiO_2_-nanotubes (SepTNT), selenium incorporated phosphate-doped TiO_2_-nanotubes with hydroxyapatite coating (SepTNT-HA), phosphate-doped TiO_2_-nanotubes (pTNT), titanium nanotubes (TNT), silver incorporated titanium phosphate-doped TiO_2_-nanotubes (AgpTNT), silver-selenium incorporated phosphate-doped TiO_2_-nanotubes (Ag_2_SepTNT) and non-modified control (Ti6Al4V).

**Figure 4:**
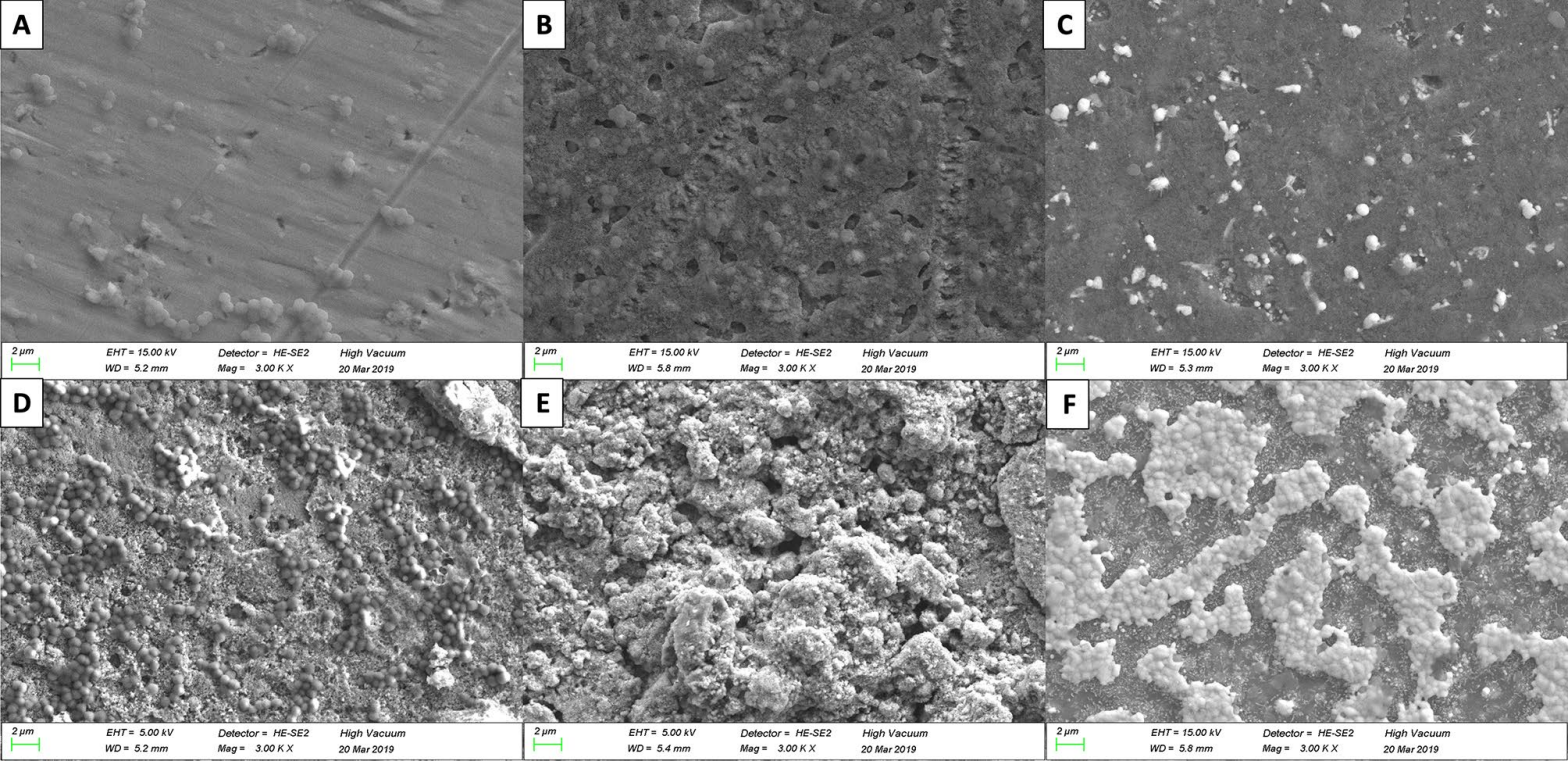
Scanning electron microscope (SEM) images of the discs’ surfaces after 24 h of incubation with *S. epidermidis*; (**A**) non-modified control shows several bacterial colonies; (**B**) With the use of Phosphate-doped TiO_2_-nanotubes (pTNT) a trend towards and increased bacterial growth; (**C**) additional selenium-incorporation (SepTNT) lead to significant reduction of bacterial colonies; (**D**) hydroxyapatite-coating (HA) was not capable in reducing bacterial growth; (**E**) in the presence of selenium in combination with HA-coating a distinct reduction of bacteria was detected; (**F**) although distinct bacterial colonies were detected at the surface of silver-selenium incorporated phosphate-doped TiO_2_-nanotubes (Ag_2_SepTNT) most of bacteria was not viable and already ingested silver-selenium particles. Only few single bacterial cells without particle ingestion were visible.

After incubating the samples with S.epidermidis and sonicating to detach bacterial cells, CFU/mL on agar plates were evaluated. A significant lower bacterial cell count could be detected for SepTNT (0.97 ± 0.18 × 10^6^ CFU/mL; *P* = 0.001), SepTNT-HA (1.2 ± 0.39 × 10^6^ CFU/mL; *P* =  0.001), AgpTNT (1.36 ± 0.42 × 10^6^ CFU/mL; *P* = 0.002) and Ag_2_SepTNT (0.999 ± 0.12 × 10^6^ CFU/mL; *P* = 0.001) compared to the non-modified control (2.2 ± 0.21 × 10^6^ CFU/mL). Figure 2 encompasses all bacterial experiments (CFU, Biofilm and fluorescence staining) of this study. Figure 3 displays results from CFU counts experiment. Bacterial cell adhesion was significantly lower in SepTNT (38 ± 12%; *P* = 0.004), pTNT (47 ± 6%, *P* = 0.040), AgpTNT (25 ± 2%; *P* < 0.001) and Ag_2_SepTNT (15 ± 5%; *P* < 0.001) compared to the non-modified control (74 ± 11%). SEM images reflect the results from the bacterial count experiments. Examples from SEM-images can be found in Fig. 4. SEM-images with Ag-coating (AgpTNT and Ag_2_SepTNT) show an incorporation of the silver and silver-selenium particles with a higher number of bacterial cells on the surface than in the CFU count. However, cells with ingested particles seemed to be non-viable anymore (Fig. 4).

**Figure 5:**
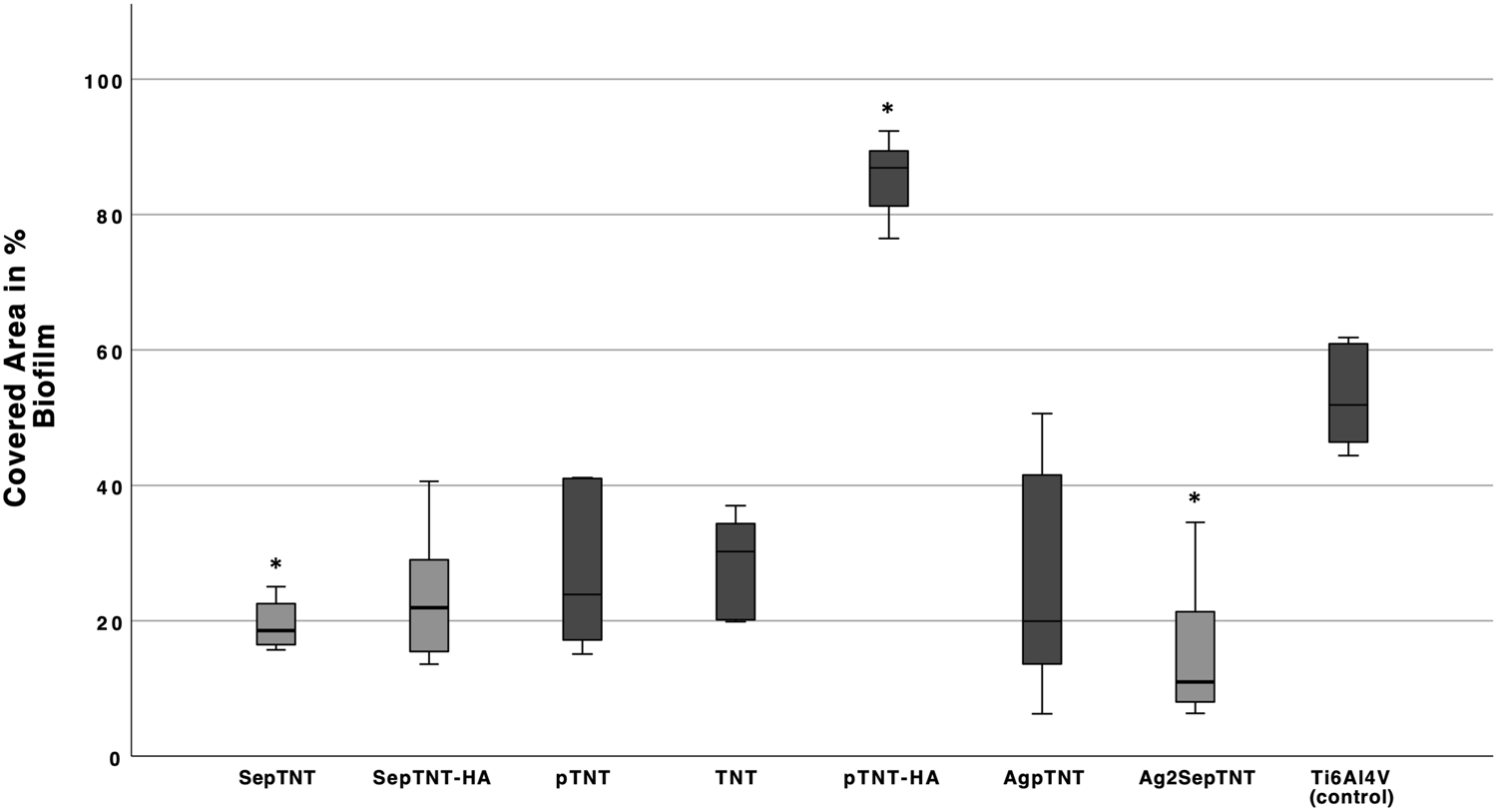
Biofilm coverage of modified and non-modified titanium discs. Selenium- and silver-selenium incorporated phosphate-doped TiO_2_-nanotubes (SepTNT, Ag_2_SepTNT) showed a significant reduction in biofilm formation whereas hydroxyapatite-coated discs with phosphate-doped TiO_2_-nanotubes showed an increased biofilm formation compared to the non-modified control.

Crystal Violet staining revealed a significant reduction in biofilm formation with SepTNT- (22 ± 3%, *P* = 0.020) and Ag_2_SepTNT discs (23 ± 11%, *P* = 0.020) compared to the non-modified control (54 ± 8%). Results of biofilm staining are presented in Fig. 5.

### Osteoblastic cell growth

**Figure 6:**
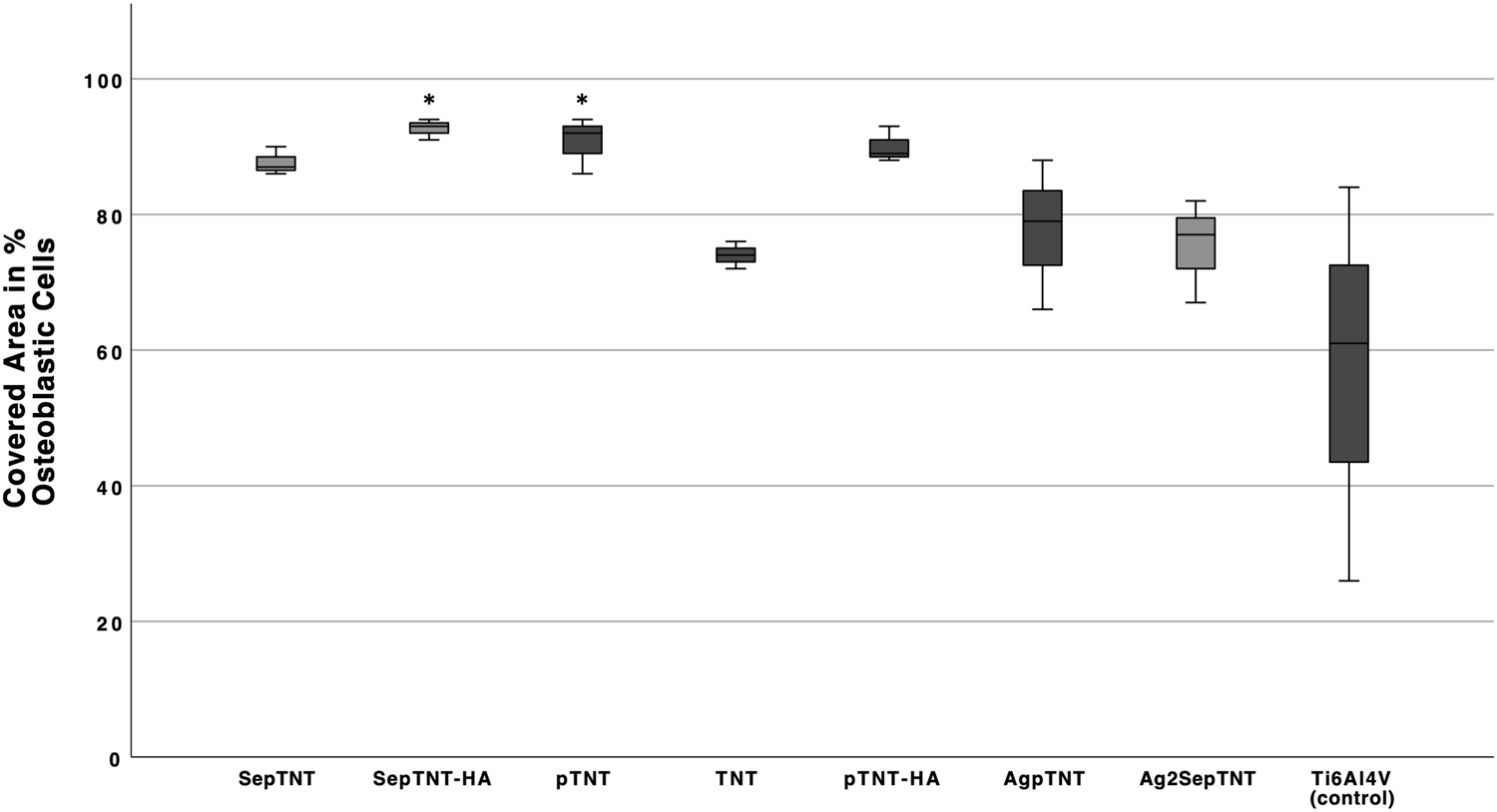
Osteoblastic cell growth on modified and non-modified titanium discs. Selenium incorporated phosphate-doped TiO_2_-nanotubes with hydroxyapatite coating (SepTNT-HA) and phosphate-doped TiO_2_-nanotubes (pTNT) showed a significant increase in osteoblastic cell growth.

**Figure 7:**
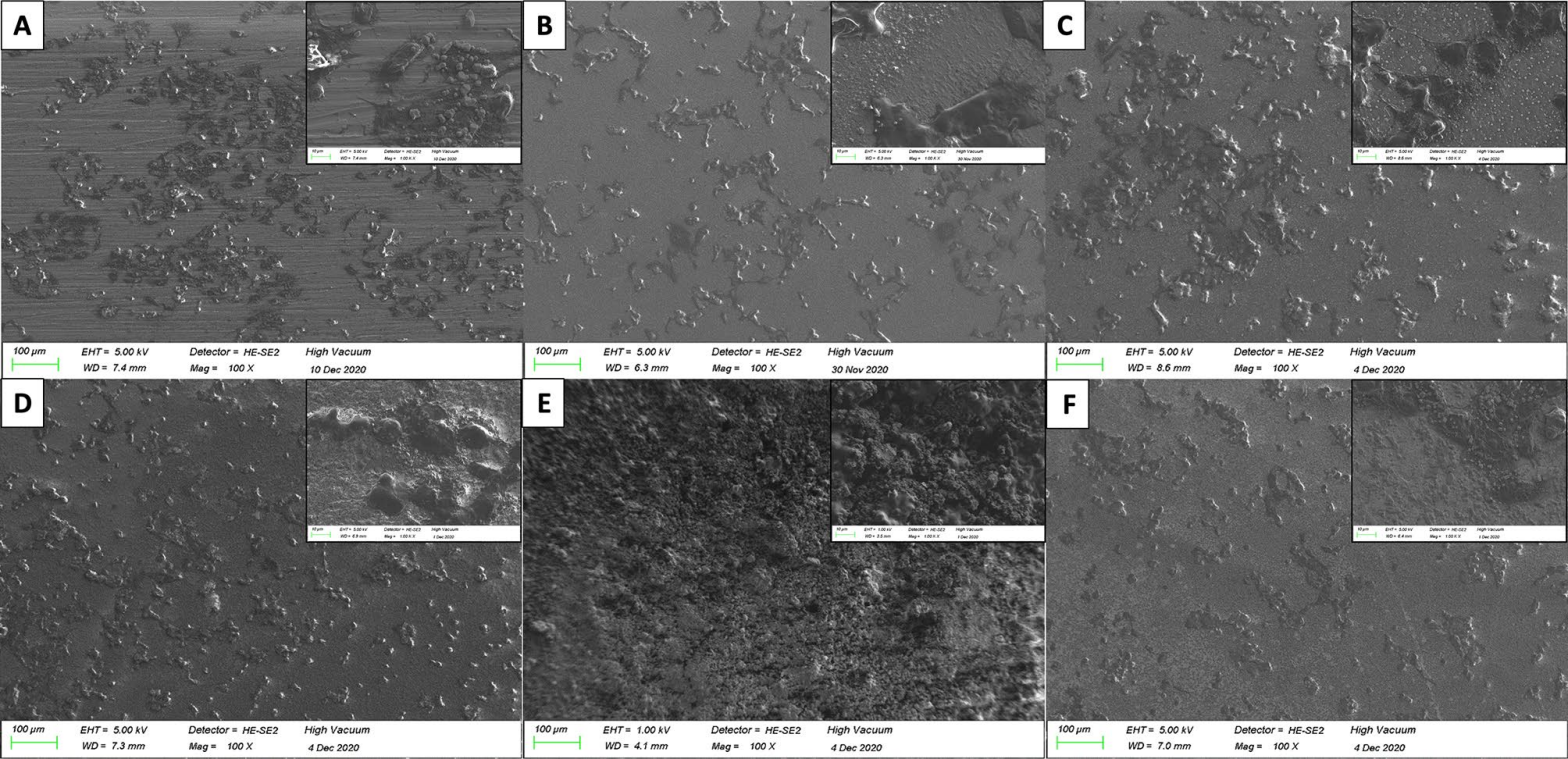
Scanning electron microscope (SEM) images with two different magnifications (100 × and 1000x) of the discs’ surfaces after 24 h of incubation with osteoblastic cell line MG-63; (**A**) non-modified control; (**B**) Samples with phosphate-doped TiO_2_-nanotubes (pTNT) seem to have more viable osteoblastic cells compared to the control; (**C**) selenium-incorporation (SepTNT) leads to a significant increase in cell density and agglomerations; (**D**) hydroxyapatite-coating (HA) on pTNT showed a more of osteoblastic cells but less agglomerations; (**E**) selenium pTNT in combination with HA showed singular osteoblastic cell formation but it appeared that less agglomeration was detected; (**F**) Ag_2_SepTNT showed less osteoblastic cell growth compared with SepTNT.

After incubating the samples with MG63-cells, discs were staining with crystal violet to measure osteoblastic cell growth. In comparison with the non-modified control, (57 ± 29%) SepTNT-HA discs (93 ± 2%; 0.027) and pTNT discs (90 ± 4%, *P* = 0.040) showed a significant higher covered area with osteoblastic MG-63 cells. No difference in osteoblastic cell growth could be detected when compared with the HA-coated discs (90 ± 3%). Figure 6 shows the coverage with MG-63 cells on the discs. SEM-images of MG-63 on the titanium surfaces are displayed on Fig. 7.

## Discussion

The need for new therapeutic strategies to prevent PJI is becoming more important as the incidence of septic complications after TJA is increasing due to a growing number of TJA and revision arthroplasties^5^. Modifications of titanium implant surfaces to enhance antimicrobial potential is one of those novel preventive approaches. In this study we report on promising results regarding the reduced growth of *S. epidermidis* on electrochemically modified titanium surfaces by using silver-selenium compounds together with titanium nanotube formation.

This study shows several limitations and our results should be put in relative perspective to these factors:

First, this study is an in-vitro study and only gives restricted reliability to translate these findings in the real clinical application setting. Especially, the osteogenic potential of the modified surfaces needs further analysis in terms of immunohistological and biomechanical evaluation in in-vivo models. Also, in order to investigate the host response to the modified implants, animal studies are needed to proceed with the translation into human clinical studies. Second, due to the high manufacturing cost of the modified discs we were not able to investigate other pathogens and more time points after incubation which might have shown different results than the ones described in this study. Third, in an in-vitro study setting it is not possible to investigate possible side-effects due to Selenium-coating of the titanium discs. However, there are reports on the use of the intravenous application of high-dose sodium-selenite in critically ill patients and no adverse side effects could be detected^30^. Nevertheless, it appears inevitable that further investigations in terms of an animal study setting are needed prior to giving further predictions for the clinical use of Selenium/nanotube surface modifications for human implants.

Nanostructuring of titanium surfaces in the shape of nanotubes has already drawn attention in the past for improving cell adhesion, growth and differentiation of bone forming cells^31–33^. And TiO_2_-nanotubes are already used on the surface of conventional cementless total knee arthroplasty systems and show improved osseointegrative behavior and antimicrobial effects^33^. Peng et al. describe in their study that the formation of nanotubes on the titanium surfaces leads to a decreased growth of *S. epidermidis*^34^. In our study, TiO_2_-nanotubes (pTNT and TNT) showed a trend towards a decreased adhesion of *S. epidermidis* compared to the non-modified titanium surface after 24 h of incubation. However, the reason why we did not see as distinct effects in bacterial cell reduction might be due to the different size of nanotubes in our study (100 nm). However, from previous works from our study group we were able to see an increased biofilm formation with tube diameters of 70 nm. A tube diameter of 100 nm showed a decrease in biofilm under SEM investigation^28^.

To enhance the antimicrobial potential of TiO_2_-nanotube surfaces, several studies have been published with additional nanotube coatings/fillings^35^. As early as 2007, Popat et al. were able to show a decreased growth of *S. epidermidis* with gentamycin-filled TiO_2_- nanotubes. Nonetheless, with the rising problem of antibiotic resistance we believe that it is of vital importance for alternative antimicrobial strategies for PJI. Therefore, the use of different antimicrobial substrates like silver or copper have been introduced as coating material. Silver has shown auspicious antimicrobial effects and is widely used in arthroplasty implants for patients with a high risk for developing a PJI like megaprosthetic and revision arthroplasty systems^26^. However, due to the toxicity^16^ a widespread use of silver as preventive agent in primary total joint replacement is not possible and is therefore only limited to revision arthroplasty and reconstruction after tumor resection. Additionally, due to the toxic effects silver coating can only be applied on implant parts that are not in direct contact to the bone^26^, In our study, the highest reduction of bacterial growth was observed with a combination of silver and selenium doped nanotubes. We speculate this might be due to underlying antimicrobial mechanism of TNT and silver-selenium deposition which creates an environment of synergistic antimicrobial effects: First, TNT are able to decrease bacterial adhesion due to its material properties (hydrophilicity, tubular structures, roughness) which prevent bacterial agglomeration while increasing osteoblastic cell growth^10^. Our study group was able to show two different mechanisms: a spontaneous catalytic formation of reactive oxygen species (ROS) in terms of H_2_O_2_ on silver-selenide surfaces in presence of oxygen due to the surface properties and the release of (toxic) silver-, copper- and selenium ions under reducing conditions^29^. Therefore, we believe that our findings are crucial to highlight the beneficial synergistic mechanisms in reducing bacterial cell growth on titanium surfaces. Additionally, these findings might also be helpful to reduce silver concentrations to lower toxic levels without impairing the antimicrobial due to the combination of selenium.

**Figure 8:**
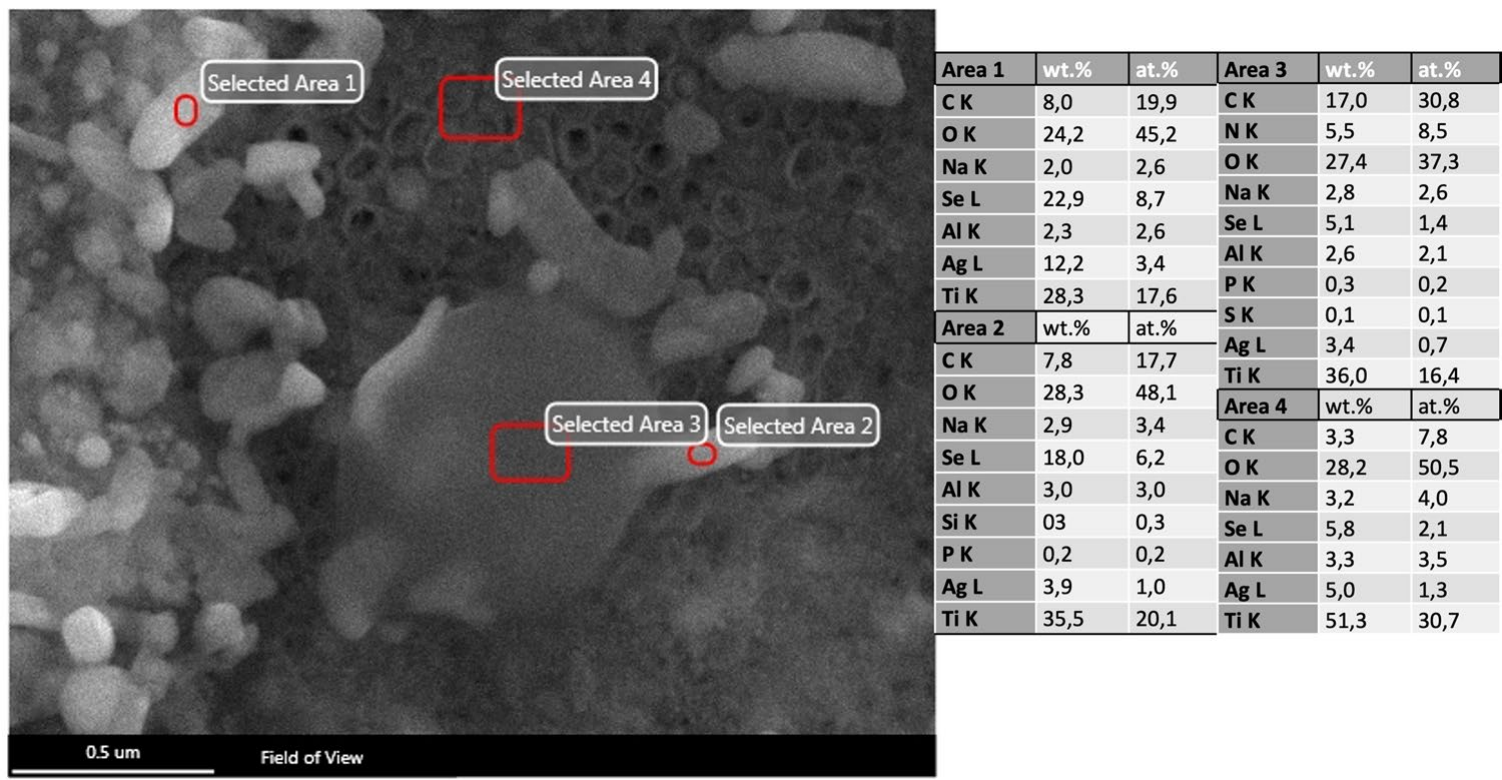
SEM-image and EDX profiles of Ag_2_SepTNT disc showing Ag_2_Se-compounds within the bacterial cells.

The use of selenium for antimicrobial orthopedic implant coating has already been described by Holinka et al.^27^. In their study, the authors found a decreased bacterial growth of S. aureus and S. epidermidis with different sodium-selenite-concentrations. Additionally, the authors describe no decrease in the growth of osteoblastic MG-63 cells. The use of the combination of TiO_2_-nanotubes and selenium has been described in a recent study by Bilek et al.^24^ and their findings are congruent to the findings of our study. However, we believe that coating surfaces by simple dipping^27^ or washing methods^24^ does not necessarily guarantee a constant and equal distribution of selenium particles on the whole surface. Therefore, our proposed method has been shown to be reliable to fill the titania nanotubes with the desired amount of selenium in a standardized way^28^. Furthermore, recent study of our group revealed that the antibacterial effect of selenides (Ag_2_Se, Cu_2_Se) could be related to both ion release and indirect oxygen reduction reaction (ORR) forming H_2_O_2_ oxidative species^29^. Interestingly, SEM images, as a qualitative marker, showed a considerable correlation with the CFU counts. However, Ag_2_SepTNT surfaces showed more bacteria on SEM images than with CFU counts detected (Fig. 4). Having a closer look at the SEM images, we found that many bacterial colonies already showed intracellular Ag_2_Se-colloids (Fig. 8). Therefore, we speculate that, similar to the bactericidal effect of silver—Ag_2_Se deteriorates the cell membrane and interacts with bacterial DNA leading to bacterial death.

Interestingly, with the HA-coated nanotubes we found an almost similar CFU count and even higher biofilm-formation compared to the unmodified control. We believe that this is due to size of the HA-particles that almost cover the orifice of the nanotubes and therefore might hinder the antimicrobial effect of the nanotubes. Due to the mechanical properties of HA, biofilm might not be as easily detached. This phenomenon was also observed and confirmed in other studies^36,37^. Additionally, HA covered the orifice of TNT which might reduce the antimicrobial potential of the TNT. Interestingly, when selenium was deposited (SepTNT-HA), antibacterial potential was increased. We therefore do not encourage the combined use of HA and TiO_2_-nanotubes with our proposed method. McEvoy et al. found similar results regarding an increased bacterial growth on HA-coated Kirschner wires compared to non-coated Ti_6_Al_4_V wires^38^.

According to our findings, the use of selenium doped TiO_2_-nanotubes does not have any influence on the growth of osteoblastic cells compared to regular titanium surfaces. There are reports that osteoblastic cell growth is enhanced with the use of nanotubes^39,40^. However, bone formation and bone-related gene expression levels were increased when diameters of approximately 70 nm were used. In our study we used 100 nm purposely in order to reduce biofilm formation. Nonetheless, we still think these findings are promising since osteoblastic cell growth is not impaired with the here presented surface modification method while enhancing the antimicrobial potential. Some studies even report on an increased osteoblast cell adhesion with selenium incorporation on the titanium surface^41^ and the use of titanium nanotubes has, as mentioned before, shown to have enhanced osseointegrative potential^31–33^. However, since different studies use different osteoblastic cell lines these findings might not be exactly comparable with each other. Also, according to our data, the formation of TiO_2_-nanotubes does not sufficiently reduce bacterial adhesion. The addition of antimicrobial agents like selenium and/or silver drastically enhances the antimicrobial potential of TiO_2_-nanotube-modified surface.

The reason why silver-selenide combined with TiO_2_-nanotubes shows the best bacterial reduction is complex and might be related to the synergistic effect of silver and selenium promoting the formation of reactive oxygen species, when in a compound. A slow release of antibacterial metal ions and the surface topography, predetermined by the nanotubes structure contribute to the observed effect. H_2_O_2_ formation and metal ions release, caused by dissolution increase the permeability of the bacterial membrane and deteriorate the bacterial DNA ultimately. The combination of selenium and silver in compounds might also have the benefit of reducing potential toxic levels of the ions released compared with using the pure metals separately.

In conclusion, in this study we show the antimicrobial and osseointegrative potential of a novel surface modification with TiO_2_-nanotubes and subsequent incorporation of additional antimicrobial agents. This leads to a significant decrease of bacterial growth on SepTNT, SepTNT-HA, AgpTNT and Ag_2_SepTNT and a significant higher area coverage with osteoblastic MG-63-cells for SepTNT-HA and pTNT surfaces in comparison with non-modified controls. These findings are of vital importance, since synergistic effects of selenium and silver together with titanium nanotubes could be detected, which can increase the antimicrobial potential of these surface modifications. Additionally, hydroxyapatite incorporated on titanium nanotube surfaces shows enhance osseointegrative potential while antibacterial effects are diminished with the addition of hydroxyapatite on nanotube surfaces. These findings are fundamental for further investigations in terms of dynamic in-vitro and in-vivo experiments to find novel therapeutic strategies to prevent PJI in the future.

## Methods

### Surface preparation and coatings

The electrochemical surface modification is based on previous work by the study group and was already described in detail^28^. Briefly, titanium discs (Ti6Al4V) with diameter of 10 mm and 1–2 mm thickness were grinded, polished, degreased and cleaned with ethanol and deionized water. The formation of nanotubes on the surface of the discs was created by anodization at a constant potential of 30 V in a two-electrode configuration with the discs as anode and a Pt foil served as counter electrode. Ethylene glycol-based electrolytes containing 10 vol% double distilled water, 0.12 M NH_4_F and 10 mM (NH_4_)NaH(PO_4_) 4H_2_O have been used. Previous investigations have shown that ethylene glycol-based electrolytes have the potential of shaping uniform nanotubes^28^. After anodization the discs were cleaned by ultrasonication in ethylene glycol and subsequently annealed in air (450 °C, 2 h) to transform TiO_2_ phase(s) into anatase to complete the nanotube- (TNT) and Phosphate-doped nanotube (pTNT) formation on the titanium surface of the discs.

Electrochemical deposition of selenium (Se), silver (Ag), silver-selenide (Ag_2_Se) in the as prepared pTNT was carried out in a three-electrode configuration. pTNT served as counter electrode and Ag/AgCI as reference electrode. Selenium was deposited by cathodic pulses in Na_2_SeO_3_-electrolyte. Silver selenide was deposited from a solution containing 0.5 M NaSCN, 5 mM AgNO_3_ ans 2.5 mM Na_2_SeO_3_. Additionally, a set of Se-pTNT and pTNT discs were additively coated with hydroxyapatite (HA) by electrochemically assisted precipitation from 2.5 mM Ca(NO3)2 4H2O and 1.5 mM (NH_4_)NaH(PO_4_) 4H_2_O with and without 2.5 mM Na_2_SeO_3_^28^. After electrochemical surface modifications, the as-prepared surfaces were used for further in-vitro testing:

Ti6Al4V (control), pTNT, TNT, pTNT-HA, SepTNT, SepTNT-HA, AgpTNT, Ag_2_SepTNT. Concentrations of Se and Ag on the surface area was determined by scanning a randomly selected area on the surface of the discs with a coupled energy-dispersive X-ray (EDX) detector, TEAM OCTANE PLUS Version 4.3. and expressed as mean percent of the component composition (weight%, wt%). Further characterization of surface properties and characteristics including elemental composition by EDX mapping, chemical composition by RAMAN spectroscopy and ion release profile using inductively coupled plasma mass spectrometry (ICP-MS have been carried out and have been previously published^28,29^.

### Preparation of bacterial cell culture

A biofilm-forming strain of *Staphylococcus epidermidis (S. epidermidis)* (DSM 3269; German Collection of Microorganisms and Cell Cultures GmBH, Leibnitz, Germany) was used in this study. Bacteria were grown overnight at 37 °C on Columbia agar plates with 5% sheep blood (Biomerieux, Craponne, France) and stored at 4 °C. For each experiment a new blood agar plate was inoculated with the strain and incubated overnight. From this plate a bacterial suspension in 0.9% saline solution with an optical density of McFarland 0.5 was used and was then diluted 1:100 in Mueller–Hinton broth (Sigma-Aldrich, St. Louis, Missuouri) (approximately 1 × 10^6^ cells/ml) for bacterial cell count and biofilm formation experiments.

### Bacterial cell count

Discs from each coating were used for the experiment twice in triplicates. The discs were put in a 24-well plate and 1 ml of the as described bacterial cell suspension was transferred in each well. The well plates were sealed and incubated at 37 °C in ambient air for 24 h. After incubation, the discs were washed twice with PBS (Sigma-Aldrich, St. Louis, Missuouri). Afterwards the discs were sonicated in PBS (Bandelin Sonorex Super RK 100) at an intensity of 44 kHz for 10 min. The resulting sonicate fluid (10 ml) was plated in 1-ml aliquots onto Columbia agar plates and again incubated at 37 °C in ambient air for 24 h. After incubation, photographs of the plates were taken and colony forming units (CFU) per milliliter (CFU/mL) were counted with ImageJ software (version 2.1.0). An additional triplicate of discs was incubated with bacterial suspension in 24-well-plates at 37 °C in ambient air for 24 h. After incubation, the discs were carefully washed in PBS and fixed with methanol. Afterwards the discs’ surfaces were analyzed again by SEM for visualization of bacterial adherence (Fig. 1).

### Bacterial cell adhesion and biofilm staining

Discs were incubated with bacterial suspension as described above. After incubation for 24 h in 37 °C the discs were carefully washed twice in distilled water. A solution of 3 μl of SYTO 9 was added to 1 ml filter-sterilized water. The discs were gently washed in PBS (3 ml, three times) and 750 μl of staining solution was added onto the disc. The staining dish was covered and the samples were incubated for 20–30 min at room temperature protected from light. After incubation, the samples were again rinsed with filter-sterilized water and observed under a fluorescence microscope (Zeiss Axioplan 2 Fluorescence Phasecontrast Microscope, Carl Zeiss, Jena, Germany) with a 10×  magnification.

Additional discs (in triplicate) were incubated again with bacterial suspension then fixed with methanol and stained with 1% crystal violet (Sigma-Aldrich, St. Louis, Missouri) for 15 min, washed again with distilled water and air dried. Afterwards, photographs of the discs were taken under standardized conditions (background, lighting, distance to lense) and calculation of the covered area was performed using ImageJ software (version 2.1.0). The staining was carried out twice in triplicates for each coating.

### Osteoblastic cell growth

To detect any influence on osteoblastic cell growth, MG-63 cells from osteoblastic cell line purchased from American Type Culture Collection (ATCC, USA, VA, CRL-1427) were cultured in 25 cm^2^ tissue culture flasks (Falcon; Thermo Fisher Scientific, Slangerup, Denmark) in Alpha-MEM (PAN-Biotech, Aidenbach, Germany) with 10% FCS in humidified atmosphere with 5% CO_2_ and incubated at 37 °C. At about 70% confluence, the cells were detached with trypsin/ EDTA (Gibco ™, Thermo Fisher Scientific, Slangerup, Denmark) at and diluted in Alpha-MEM + FCS to obtain the final concentration of 1.5 × 10^5^ cells/ml. 1 ml of the solution was inoculated on each titanium disc and incubated for 24 h in a humidified atmosphere at 37 °C and 5% CO_2_. After incubation discs were washed in distilled water and fixed with methanol for 10 min. The discs were washed in distilled water, stained with crystal violet for 15 min, washed again with distilled water and air dried. Afterwards, photographs of the discs were taken under standardized conditions (background, lighting, distance to lense) and calculation of the covered area was performed using ImageJ software (version 2.1.0). The staining was carried out twice in triplicates for each coating.

### Statistical analysis

Results are presented as mean and standard deviation (SD). Numerical variables (CFU/mL, covered area in %) were analyzed using a one-way analysis of variances (ANOVA) and compared using a Tukey-HSD post-hoc test. Results were considered statistically significant with a *P* value < 0.05. Statistical analysis was performed using SPSS 26.0.0.1 (SPSS Inc. IBM, Chicago, USA).

### Statements and declarations

All authors declare no competing interest related to this study. R.W. receives royalties from DePuy Synthes (Warsaw, IN, USA) and Stryker (Kalamazoo, MI, USA) outside the submitted work.

Each author certifies that his or her institution approved the study protocol for this investigation and that all investigations were conducted in conformity with ethical principles of research.

## Supplementary Information

Supplementary Information.

## Data Availability

All data generated or analyzed during this study are included in this published article and in its supplementary information files.
