# Supplementary material for: Antimicrobial potential and osteoblastic cell growth on electrochemically modified titanium surfaces with nanotubes and selenium or silver incorporation
Source: Sci Rep. 2022 May 18;12:8298. doi: 10.1038/s41598-022-11804-6 (PMC9117198; doi:10.1038/s41598-022-11804-6)
Supplement: Supplementary file 1 — Supplementary Information. [file 41598_2022_11804_MOESM1_ESM.pdf]

group(1=SepTNT, :	CFU/ml	with Seleniur	TNT/SeTNT/control
1	890000	1	1
1	850000	1	1
1	905000	1	1
1	1087210	1	1
1	1287310	1	1
1	827080	1	1
2	1860000	1	1
2	925000	1	1
2	745000	1	1
2	1047190	1	1
2	1320660	1	1
2	1354010	1	1
3	2795000	0	0
3	2980000	0	0
3	2170000	0	0
3	1340670	0	0
3	1427380	0	0
3	1574120	0	0
4	1650000	0	0
4	2650000	0	0
4	1465000	0	0
4	1233950	0	0
4	1860930	0	0
4	1040520	0	0
5	1715000	0	0
5	2300000	0	0
5	1730000	0	0
5	1920960	0	0
5	2214440	0	0
5	1714190	0	0
6	1935000	0	0
6	1355000	0	0
6	835000	0	0
6	1667500	0	0
6	1440720	0	0
6	920460	0	0
7	870000	1	1
7	945000	1	1
7	960000	1	1
7	920460	1	1
7	1113890	1	1
7	1187260	1	1
10	1045000	0	2
10	1335000	0	2
10	1190000	0	2
10	1567450	0	2
10	1754210	0	2
10	1734200	0	2

1=SepTNT, 2=SeHap, 3=pTNT, 4=TNT, 5=HaP, 6=AgpTNT, 7=Ag2SepTNT, 10=non-modified

group(1=SepT	covered area	TNT/TNT-Se/control
3	94	0
3	86	0
3	92	0
1	87	1
1	90	1
1	86	1
2	91	1
2	93	1
2	94	1
4	74	0
4	76	0
4	72	0
5	93	0
5	89	0
5	88	0
6	66	0
6	88	0
6	79	0
7	67	1
7	82	1
7	77	1
10	84	2
10	26	2
10	61	2

group(1=SepT sonicated		covered area	TNT/TNT-Se/control/HaP
1	1	15,71	1
1	1	17,73	1
1	1	16,48	1
2	1	15,47	1
2	1	13,6	1
2	1	29,01	1
3	1	17,17	0
3	1	15,09	0
3	1	22,33	0
4	1	19,87	0
4	1	20,15	0
4	1	33,06	0
5	1	76,45	0
5	1	81,26	0
5	1	88,24	0
6	1	19,05	0
6	1	6,25	0
6	1	20,83	0
7	1	6,32	1
7	1	8,79	1
7	1	8	1
10	1	44,4	2
10	1	49,4	2
10	1	60,9	2
1	0	19,38	1
1	0	25,05	1
1	0	22,54	1
2	0	20,69	1
2	0	23,2	1
2	0	40,6	1
3	0	25,46	0
3	0	41,15	0
3	0	41,02	0
4	0	34,36	0
4	0	27,42	0
4	0	37,01	0
5	0	89,4	0
5	0	85,53	0
5	0	92,34	0
6	0	50,6	0
6	0	13,63	0
6	0	41,53	0
7	0	21,35	1
7	0	34,55	1
7	0	13,11	1
10	0	54,34	2
10	0	46,39	2
10	0	61,82	2
